# Identification of cross-talk between m^6^A and 5mC regulators associated with onco-immunogenic features and prognosis across 33 cancer types

**DOI:** 10.1186/s13045-020-00854-w

**Published:** 2020-03-18

**Authors:** Yu-Tong Chen, Jia-Yi Shen, Dong-Ping Chen, Chen-Fei Wu, Rui Guo, Pan-Pan Zhang, Jia-Wei Lv, Wen-Fei Li, Zi-Xian Wang, Yu-Pei Chen

**Affiliations:** 1 State Key Laboratory of Oncology in South China, Collaborative Innovation Center for Cancer Medicine, Guangdong Key Laboratory of Nasopharyngeal Carcinoma Diagnosis and Therapy, Sun Yat-sen University Cancer Center, Guangzhou, 510060 People’s Republic of China; 2 Department of Medical Oncology, The Third Affiliated Hospital of Sun Yat-sen University, Sun Yat-Sen University, Guangzhou, 510632 People’s Republic of China; 3 School of Medicine, Jinan University, Guangzhou, 510632 People’s Republic of China; 4 MOE Key Laboratory of Gene Function and Regulation, School of Life Sciences, Sun Yat-sen University, Guangzhou, 510275 People’s Republic of China

**Keywords:** m^6^A regulators, 5mC regulators, Pan-cancer analyses, Genomic alterations, Tumor microenvironment, Survival

## Abstract

Methylation of RNA and DNA, notably in the forms of N6-methyladenosine (m^6^A) and 5-methylcytosine (5mC) respectively, plays crucial roles in diverse biological processes. Currently, there is a lack of knowledge regarding the cross-talk between m^6^A and 5mC regulators. Thus, we systematically performed a pan-cancer genomic analysis by depicting the molecular correlations between m^6^A and 5mC regulators across ~ 11,000 subjects representing 33 cancer types. For the first time, we identified cross-talk between m^6^A and 5mC methylation at the multiomic level. Then, we further established m^6^A/5mC epigenetic module eigengenes by combining hub m^6^A/5mC regulators and informed a comprehensive epigenetic state. The model reflected status of the tumor-immune-stromal microenvironment and was able to predict patient survival in the majority of cancer types. Our results lay a solid foundation for epigenetic regulation in human cancer and pave a new road for related therapeutic targets.

To the Editor,

Nucleotide methylation, notably in the forms of 5-methylcytosine (5mC) in DNA and N6-methyladenosine (m^6^A) in mRNA, carries important information for gene regulation [1]. Recent research advances highlight the biological importance of m^6^A methylation as a dynamic and reversible post-transcriptional modification [2]. 5mC DNA methylation, a conserved epigenetic modification along with m^6^A RNA modification, also plays critical roles in fundamental biological processes [3, 4]. In addition, recent studies have identified 5mC methylation as a modulator of alternative mRNA splicing at the post-transcriptional level [5, 6]. Although Zhou and colleagues [7] established a molecular link between 5mC DNA methylation and m^6^A mRNA methylation during fruit ripening, the potential cross-talk still remains uncharacterized in human cancers.

To address this issue, we curated a catalog of 20 and 21 genes that function mainly as regulators of RNA and DNA methylation, respectively (Fig. 1a). The genome-wide omics data comprising of 11,080 human samples across 33 cancer types from the The Cancer Genome Atlas (TCGA) were obtained for analyses (please see Methods and Table S1). First, most of the m^6^A and 5mC regulators were found to exhibit comparable expression levels across the 33 cancer types (Supplementary Fig. S1). Basing on the Gene Set Cancer Analysis (GSCA) web server [8], we further assessed the gene set differential expression profiles among 14 cancer types with available paired tumor-normal tissue expression data. Across multiple cancer types, the differentially expressed genes (upregulated or downregulated) included both m^6^A and 5mC regulators (Supplementary Fig. S2). Then, we investigated the mutation frequencies of the m^6^A and 5mC regulators. Intriguingly, m^6^A and 5mC regulators exhibited comparable levels of mutation frequency, and significant co-occurrences of genetic alterations were observed between the two regulators (Fig. 1b). Our results showed correlated expression patterns for genes within the same regulator class and even high correlations between the expression of m^6^A and 5mC regulators (Fig. 1b). Moreover, these m^6^A and 5mC regulators interacted with one another frequently in protein-protein interaction networks (Fig. 1d).

**Fig. 1:**
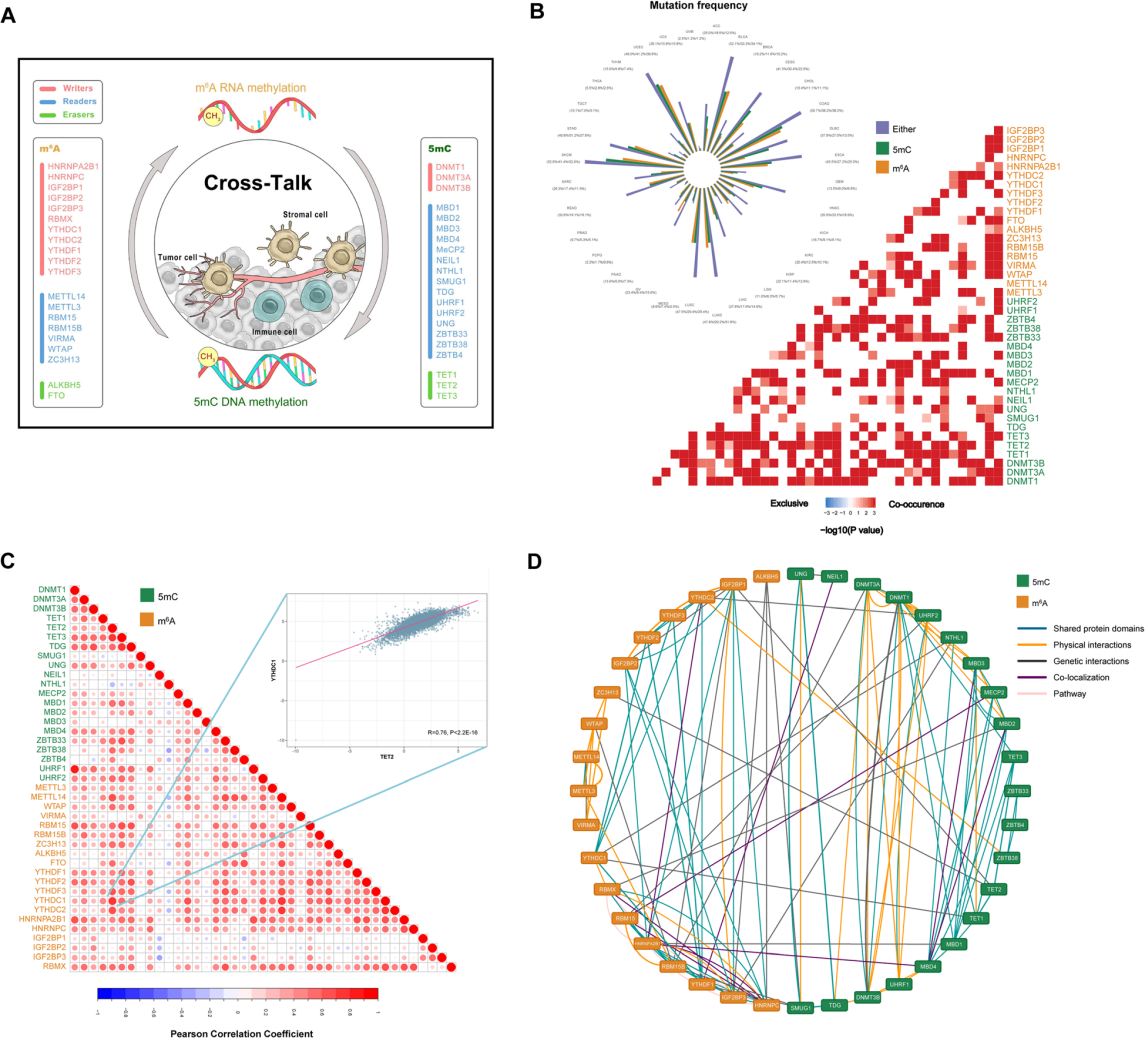
Cross-talk identified among the m^6^A and 5mC regulators. **a** m^6^A and 5mC regulator genes and a diagram of their potential cross-talk. **b** Correlations between the expression of m^6^A and 5mC regulators. The scatter plot shows the strong positive correlation between YTHDC1 and TET2. The Pearson correlation coefficients (R) are shown. **c** Co-occurrence of genetic alterations in m^6^A and 5mC regulators. The log2 (odds ratio) is colored as a heat map. The Nightingale rose diagram shows the mutation frequency distribution of m^6^A and 5mC regulators across different cancer types. **d** Protein-protein interactions among the m^6^A and 5mC regulators based on the GeneMANIA database

To identify the hub regulators involved in RNA and DNA methylation, we then applied weighted gene coexpression network analysis (WGCNA) to determine the hub genes in m^6^A and 5mC regulators (Fig. 2a). Strikingly, the number of hub m^6^A regulators was highly correlated with that of hub 5mC regulators in different cancer types (*R* = 0.84; Fig. 2b), which may be explained by the cross-talk. We then combined the hub m^6^A/5mC genes to develop an epigenetic module eigengene (EME), which may reflect both the pre- and post-transcriptional modification statuses. Next, we examined the correlation between EMEs and the activity of hallmark oncogenic pathways (Fig. 2c). Interestingly, our results indicate that high expression of the EME may reflect a highly proliferative and aggressive status in the majority of tumors. In addition, we applied GSCA [8] to analyze the effect (activation or inhibition) of m^6^A/5mC regulators on cancer-related pathways and confirmed that the m^6^A and 5mC regulators may be functionally related (Supplementary Fig. S3).

**Fig. 2:**
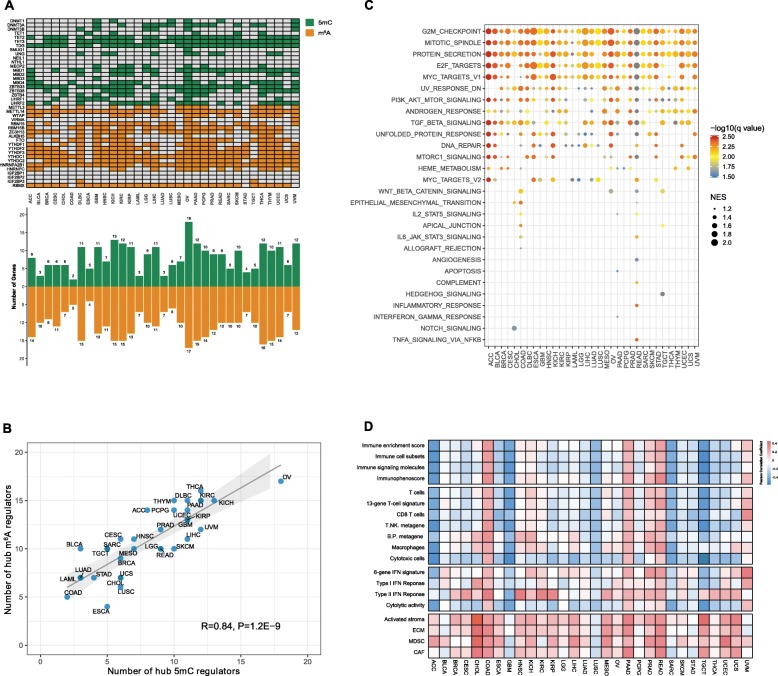
Development and characterization of the m^6^A/5mC epigenetic module eigengenes (EMEs). **a** Module membership-based hub m^6^A and 5mC regulators across 33 cancer types. The lower panel shows the number of hub m^6^A and 5mC regulators in each cancer type. **b** Correlations between the number of hub m^6^A regulators and the number of hub 5mC regulators. The Pearson correlation coefficients (R) are shown. **c** Gene set enrichment analysis (GSEA) results (normalized enrichment scores [NES] and *q* values) regarding the hallmark oncogenic pathways for EME^high^ versus EME^low^ subgroups across 33 cancer types. Enrichment score terms with an FDR < 0.05 are shown. **d** Heatmap showing the Pearson correlation coefficients between the EMEs and immuno-stromal signatures across 30 cancer types. Diffuse large B cell lymphoma (DLBC), acute myeloid leukemia (LAML), and thymoma (THYM) were excluded, as they mainly consist of immune cells

In addition to the tumor compartments, we further investigated the associations between the EME and immuno-stromal signatures representing different statuses of the immune and stromal cells (Table S2) across cancer types. In general, relatively low expression of inflammatory markers and low infiltration of immune cells were observed in the EME^high^ versus EME^low^ subgroups across cancer types (Fig. 2d). Interestingly, the high enrichment of stromal-related signatures was observed in the EME^high^ subgroups in almost all cancer types, indicating that hub m^6^A/5mC regulators may generally be involved in stroma activation (Fig. 2d).

Finally, we assessed the prognostic value of the EME in various types of cancers. We found that the EME showed oncogenic features in most cancer types, with overall survival (OS) hazard ratios larger than one (Supplementary Fig. S4a–c). Of these, high expression of the EME was significantly associated with unfavorable OS in cancer types such as KICH, ACC, and LGG (Supplementary Fig. S4a, b). Among HNSC, KIRC, and READ, improved survival was observed in the EME^high^ versus EME^low^ groups (Supplementary Fig. S4a, c).

In summary, to our best knowledge, this is the first study suggesting potential cross-talk between m^6^A and 5mC regulators in human cancers. This study provides essential insights into epigenetic regulation in cancer and paves new ways for related therapeutic targets.

## Supplementary information

**Additional file 1: Figure S1.** Gene expression profile of m^6^A/5mC regulators across 33 cancer types. Pan-cancer normalized RNA-Seq by Expectation-Maximization (RSEM) data were used. For a given m^6^A/5mC regulators in a given cancer type, the non-scaled median expression level is presented.

**Additional file 2: Figure S2.** Gene set differential expression profile of m^6^A/5mC regulators among 14 cancer types with available paired tumor-normal tissue expression data calculated by Gene Set Cancer Analysis (GSCA).

**Additional file 3: Figure S3.** Heatmap showing percentage of cancers in which a pathway may be activated (red) or inhibited (blue) by the m^6^A/5mC regulators calculated by Gene Set Cancer Analysis (GSCA). Reverse phase protein array (RPPA) data of 32 cancer types from The Cancer Proteome Atlas (TCPA) are used for the calculation; acute myeloid leukemia (LAML) is not included. A total of 10 cancer related pathways are included (i.e., TSC/mTOR, RTK, RAS/MAPK, PI3K/AKT, Hormone ER, Hormone AR, EMT, DNA Damage Response, Cell Cycle, and Apoptosis pathways), and only m^6^A/5mC regulators that have function (activate or inhibit) in at least five cancer types are shown by GSCA.

**Additional file 4: Figure S4.** Clinical relevance of the EMEs across 33 cancer types. **a** Forest plots showing the hazard ratios (HRs; squares) and 95% confidence intervals (CIs; horizontal ranges) of overall survival (OS) across 33 cancer types. Significant results are indicated by red (unfavorable prognosticators) or blue (favorable prognosticators) squares. **b** Kaplan-Meier plots showing unfavorable OS in the EME^high^ versus EME^low^ groups for KICH, ACC, LGG, CESC, SARC, LIHC, BRCA, and LUAD. *P* values for the two-sided log-rank test are shown. **c** Kaplan-Meier plots showing improved OS in the EME^high^ versus EME^low^ groups for HNSC, KIRC, and READ. *P* values for the two-sided log-rank test are shown.

**Additional file 5.** Materials and Methods.

**Additional file 6: Table S1.** Details of the 33 cancer types from the TCGA.

**Additional file 7: Table S2.** Immuno-stromal signatures used in the current study.

## Data Availability

The datasets used in this study are publicly available. All other relevant data and R and other custom scripts are available upon request.

## Abbreviations

5mC: 5-Methylcytosine
ACC: Adrenocortical carcinoma
BRCA: Breast cancer
CESC: Cervical squamous cell carcinoma and endocervical adenocarcinoma
CNV: Copy number variation
EME: Epigenetic module eigengene
GSEA: Gene set enrichment analysis
HNSC: Head and neck squamous carcinoma
KICH: Kidney chromophobe
KIRC: Kidney renal clear cell carcinoma
KIRP: Kidney renal papillary cell carcinoma
LGG: Brain low-grade glioma
LIHC: Liver hepatocellular carcinoma
LUAD: Lung adenocarcinoma
m^6^A: Methylation of N6 adenosine
OS: Overall survival
PCPG: Pheochromocytoma and paraganglioma
READ: Rectal adenocarcinoma
SARC: Sarcoma
SKCM: Skin cutaneous melanoma
TCGA: The Cancer Genome Atlas
THCA: Thyroid cancer
UCEC: Uterine corpus endometrial carcinoma
UVM: Uveal melanoma
WGCNA: Weighted gene coexpression network analysis
