# Supplementary figures and images for: Identification of cross-talk between m6A and 5mC regulators associated with onco-immunogenic features and prognosis across 33 cancer types
Source: J Hematol Oncol. 2020 Mar 18;13:22. doi: 10.1186/s13045-020-00854-w (PMC7081591; doi:10.1186/s13045-020-00854-w)

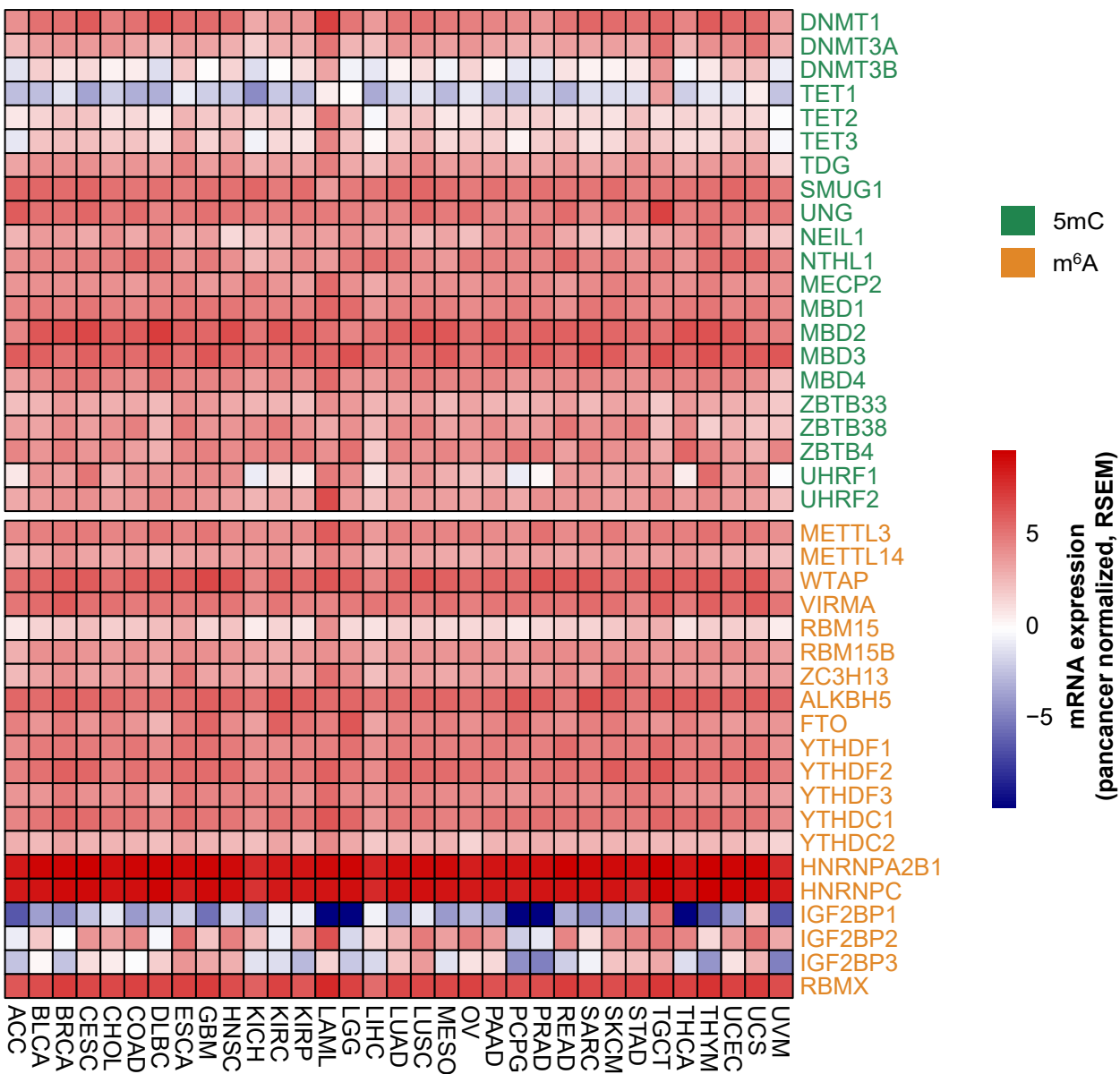

Supplement: Supplementary file 1 — Additional file 1: Figure S1. Gene expression profile of m6A/5mC regulators across 33 cancer types. Pan-cancer normalized RNA-Seq by Expectation-Maximization (RSEM) data were used. For a given m6A/5mC regulators in a given cancer type, the non-scaled median expression level is presented. [file 13045_2020_854_MOESM1_ESM.pdf]

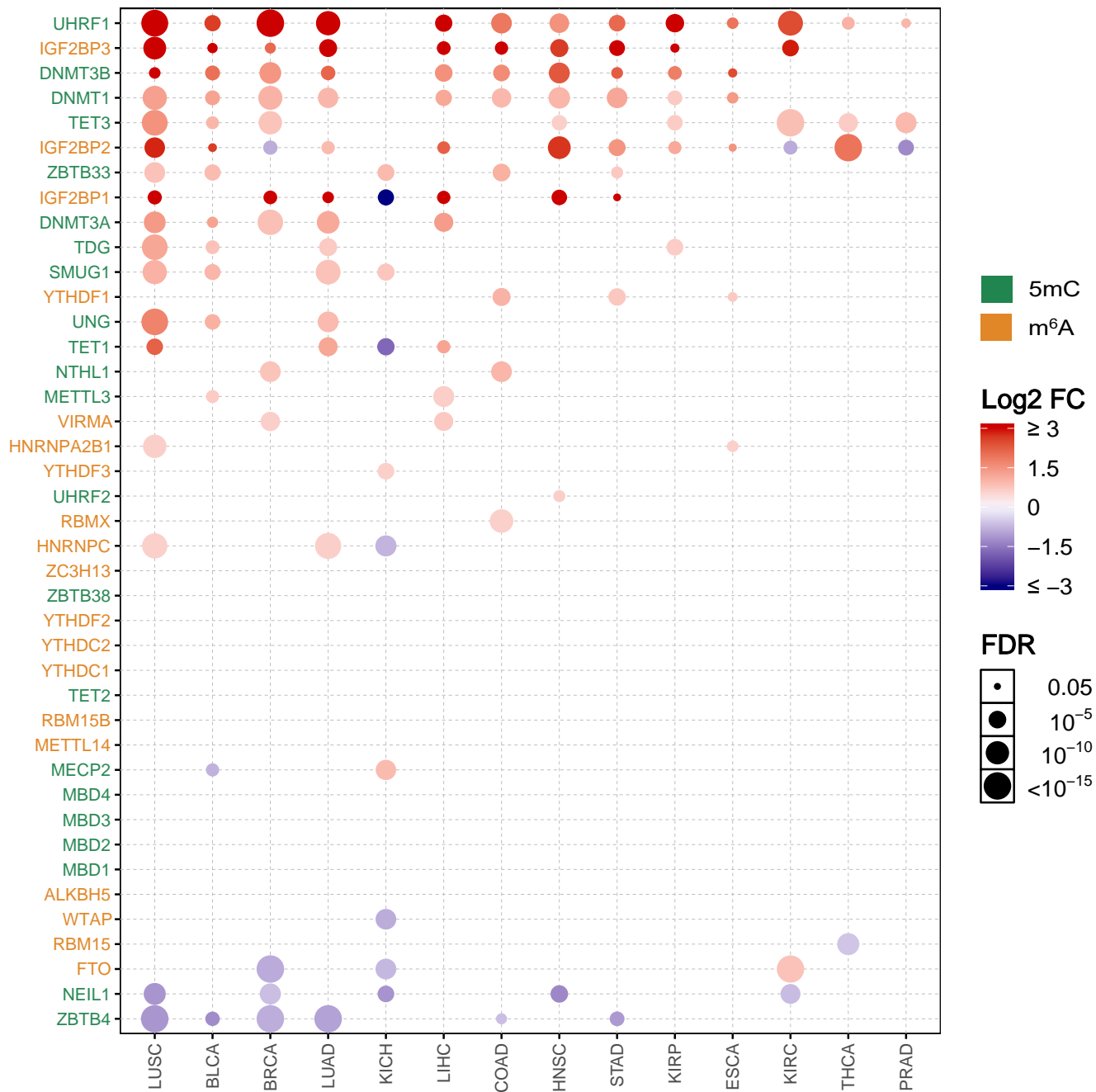

Supplement: Supplementary file 2 — Additional file 2: Figure S2. Gene set differential expression profile of m6A/5mC regulators among 14 cancer types with available paired tumor-normal tissue expression data calculated by Gene Set Cancer Analysis (GSCA). [file 13045_2020_854_MOESM2_ESM.pdf]

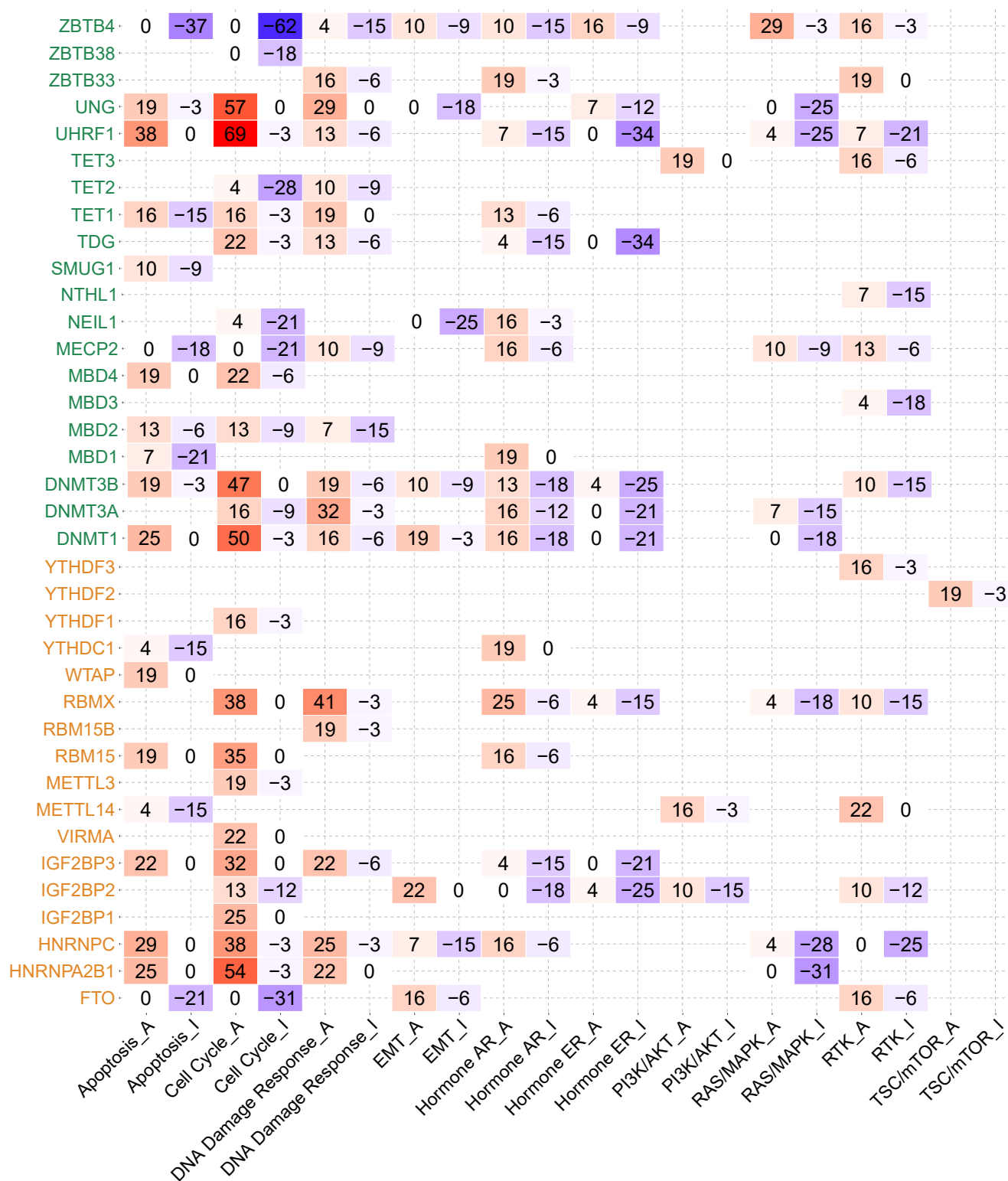

Pathway (A:Activate; I:Inhibit)

5mC  
m<sup>6</sup>A

Percent

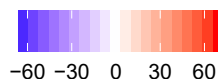

Supplement: Supplementary file 3 — Additional file 3: Figure S3. Heatmap showing percentage of cancers in which a pathway may be activated (red) or inhibited (blue) by the m6A/5mC regulators calculated by Gene Set Cancer Analysis (GSCA). Reverse phase protein array (RPPA) data of 32 cancer types from The Cancer Proteome Atlas (TCPA) are used for the calculation; acute myeloid leukemia (LAML) is not included. A total of 10 cancer related pathways are included (i.e., TSC/mTOR, RTK, RAS/MAPK, PI3K/AKT, Hormone ER, Hormone AR, EMT, DNA Damage Response, Cell Cycle, and Apoptosis pathways), and only m6A/5mC regulators that have function (activate or inhibit) in at least five cancer types are shown by GSCA. [file 13045_2020_854_MOESM3_ESM.pdf]

**A**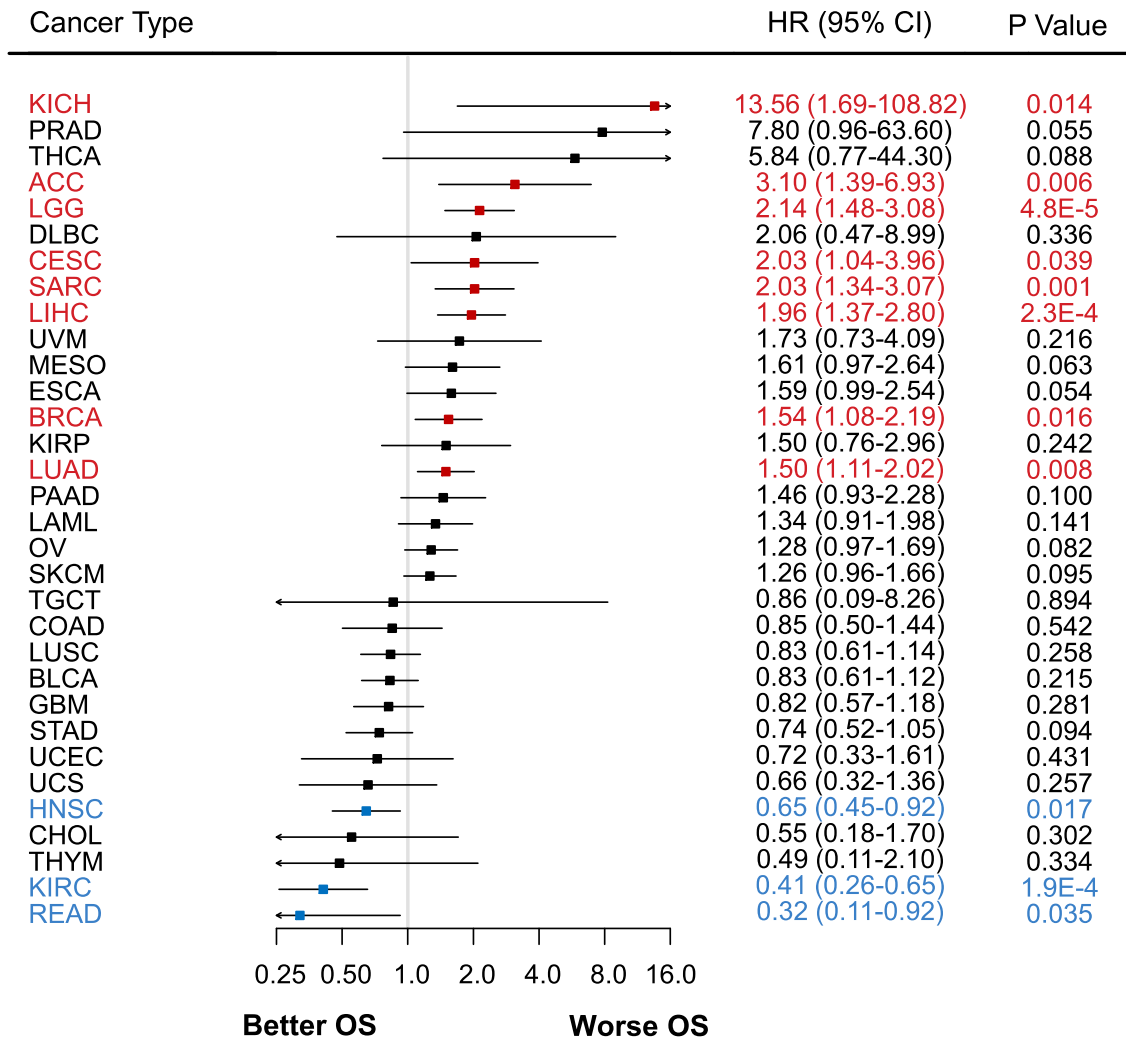**B**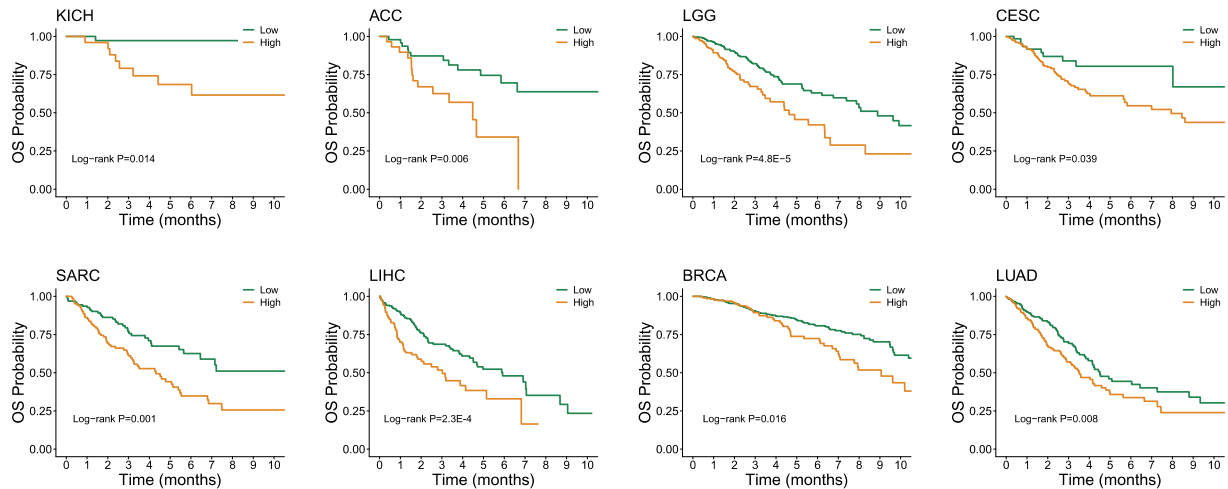**C**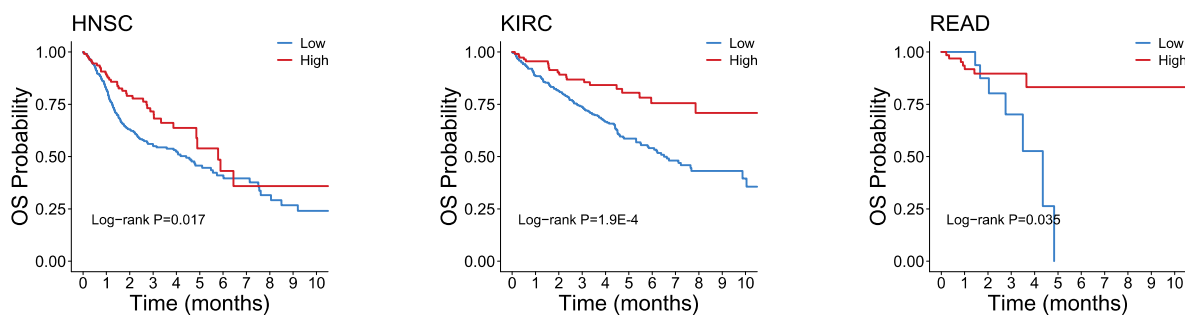

Supplement: Supplementary file 4 — Additional file 4: Figure S4. Clinical relevance of the EMEs across 33 cancer types. a Forest plots showing the hazard ratios (HRs; squares) and 95% confidence intervals (CIs; horizontal ranges) of overall survival (OS) across 33 cancer types. Significant results are indicated by red (unfavorable prognosticators) or blue (favorable prognosticators) squares. b Kaplan-Meier plots showing unfavorable OS in the EMEhigh versus EMElow groups for KICH, ACC, LGG, CESC, SARC, LIHC, BRCA, and LUAD. P values for the two-sided log-rank test are shown. c Kaplan-Meier plots showing improved OS in the EMEhigh versus EMElow groups for HNSC, KIRC, and READ. P values for the two-sided log-rank test are shown. [file 13045_2020_854_MOESM4_ESM.pdf]
