# Supplementary material for: Identification of cross-talk between m6A and 5mC regulators associated with onco-immunogenic features and prognosis across 33 cancer types
Source: J Hematol Oncol. 2020 Mar 18;13:22. doi: 10.1186/s13045-020-00854-w (PMC7081591; doi:10.1186/s13045-020-00854-w)
Supplement: Supplementary file 5 — Additional file 5. Materials and Methods. [file 13045_2020_854_MOESM5_ESM.docx]

**Materials and Methods**

**Collection of** **m^6^A/5mC regulators and datasets studied**

From published studies (1-4), we collected a total of 20 m^6^A and 21 5mC regulators. We analyzed the genome-wide omics data from a total of 11,080 human samples across 33 cancer types from The Cancer Genome Atlas (TCGA) (<http://cancergenome.nih.gov/>); the cancer types included are summarized in Table S1.

**Genomic, transcriptomic, and clinical data analyzed across cancer types**

The MC3 somatic mutation data and Level 3 RNA-Seq data, represented as RNA-Seq by Expectation-Maximization (RSEM) data normalized within each sample to the upper quartile of total reads, were downloaded from the UCSC Xena browser (<http://xena.ucsc.edu/>). In addition, we identified the protein-protein interactions among m^6^A and 5mC regulators based on the GeneMANIA interaction database (<http://genemania.org>) (5), and the interactions were visualized with Cytoscape (6). The clinical information for patients was downloaded from the TCGA via the R package *TCGAbiolinks*.

**Identification of** **hub** **m^6^A/5mC regulators based on the** **topology of the coexpression networks**

To identify hub m^6^A and 5mC regulators for each cancer type, we introduced the concept of “module” from the weighted gene coexpression network analysis (WGCNA) algorithm and treated the 41 m^6^A and 5mC regulators as a module (7). The overall expression level of the module was summarized as the module eigengene by the *moduleEigengenes* function in the R package *WGCNA*. We further calculated the module membership (i.e., module eigengene-based intramodular connectivity) as the correlation between the expression value of a given m^6^A/5mC regulator and the module eigengene. Hub m^6^A/5mC regulators were then defined as those that achieved a module membership greater than 0.7. The summary expression level of the identified hub m^6^A/5mC regulators was again calculated as epigenetic module eigengenes (EMEs) for each cancer type.

**Gene set enrichment analysis (GSEA)**

Predominantly, pathway analyses were performed to evaluate the 50 hallmark oncogenic pathways (8). The hallmark gene set was downloaded from the MSigDB database of the Broad Institute. Then, we applied GSEA with standard settings as implemented in the R package *clusterProfiler* to assign pathway activity estimates to each sample. Enrichment *P* values were based on 10,000 permutations and subsequently adjusted for multiple testing using the Benjamini-Hochberg procedure to control the FDR (9).

**Gene Set Cancer Analysis (GSCA)**

On the basis of the GSCA web server (10), we assessed the gene set differential expression profiles among 14 cancer types with available paired tumor-normal tissue expression data. We also applied GSCA to analyze the effect (activation or inhibition) of m^6^A/5mC regulators on cancer related pathways. In GSCA, reverse phase protein array (RPPA) data of 32 cancer types from The Cancer Proteome Atlas (TCPA) are used for the analysis; acute myeloid leukemia (LAML) is not included. A total of 10 cancer related pathways are included (i.e., TSC/mTOR, RTK, RAS/MAPK, PI3K/AKT, Hormone ER, Hormone AR, EMT, DNA Damage Response, Cell Cycle, and Apoptosis pathways), and only m^6^A/5mC regulators that have function (activate or inhibit) in at least five cancer types are shown by GSCA.

**Immuno-oncologic signatures analyzed**

Previously reported immuno-stromal signatures (11-22) representing different statuses of the tumor, immune, and stromal compartments (Table S2) were used to characterize the high versus low EME expression subgroups across cancer types. GSVA (23), with standard settings as implemented in the R package *GSVA*, was used to calculate the single-sample GSEA score for the gene signatures. GSVA is a nonparametric, unsupervised method for estimating variations in gene set enrichment through the samples of an expression dataset. To identify the m^6^A regulators that were correlated with the activation or inhibition of the pathways, we calculated the Pearson correlation coefficients between the expression of the EMEs and immune signatures.

**Clinical relevance of the EMEs across 33 cancer types**

To explore whether the expression of EMEs was associated with patient survival in different cancer types, we divided the patients into two groups based on the optimal cutoff value for the EMEs, which achieved maximum log-rank statistics; this was performed using the *surv_cutpoint* function of the R package *survminer*. A two-sided log-rank test was used to examine the difference in survival rates between the two groups. This process was performed by the *survdiff* function implemented in the R package *survival*. *P* values <0.05 were considered significant.

**Supplementary references**

1. Smith ZD, Meissner A. DNA methylation: roles in mammalian development. Nature reviews Genetics. 2013;14(3):204-20.

2. Meng H, Cao Y, Qin J, Song X, Zhang Q, Shi Y, et al. DNA methylation, its mediators and genome integrity. International journal of biological sciences. 2015;11(5):604-17.

3. Chen K, Zhao BS, He C. Nucleic Acid Modifications in Regulation of Gene Expression. Cell chemical biology. 2016;23(1):74-85.

4. Li Y, Xiao J, Bai J, Tian Y, Qu Y, Chen X, et al. Molecular characterization and clinical relevance of m(6)A regulators across 33 cancer types. Molecular cancer. 2019;18(1):137.

5. Franz M, Rodriguez H, Lopes C, Zuberi K, Montojo J, Bader GD, et al. GeneMANIA update 2018. Nucleic acids research. 2018;46(W1):W60-w4.

6. Shannon P, Markiel A, Ozier O, Baliga NS, Wang JT, Ramage D, et al. Cytoscape: a software environment for integrated models of biomolecular interaction networks. Genome Res. 2003;13(11):2498-504.

7. Langfelder P, Horvath S. WGCNA: an R package for weighted correlation network analysis. BMC bioinformatics. 2008;9:559.

8. Subramanian A, Tamayo P, Mootha VK, Mukherjee S, Ebert BL, Gillette MA, et al. Gene set enrichment analysis: a knowledge-based approach for interpreting genome-wide expression profiles. Proc Natl Acad Sci U S A. 2005;102(43):15545-50.

9. Benjamini Y, Hochberg YJJotRSS. Controlling the False Discovery Rate: A Practical and Powerful Approach to Multiple Testing. 1995;57(1):289-300.

10. Liu CJ, Hu FF, Xia MX, Han L, Zhang Q, Guo AY. GSCALite: a web server for gene set cancer analysis. Bioinformatics. 2018;34(21):3771-2.

11. Yoshihara K, Shahmoradgoli M, Martinez E, Vegesna R, Kim H, Torres-Garcia W, et al. Inferring tumour purity and stromal and immune cell admixture from expression data. Nature communications. 2013;4:2612.

12. Cancer Genome Atlas N. Genomic Classification of Cutaneous Melanoma. Cell. 2015;161(7):1681-96.

13. Charoentong P, Finotello F, Angelova M, Mayer C, Efremova M, Rieder D, et al. Pan-cancer Immunogenomic Analyses Reveal Genotype-Immunophenotype Relationships and Predictors of Response to Checkpoint Blockade. Cell reports. 2017;18(1):248-62.

14. Bindea G, Mlecnik B, Tosolini M, Kirilovsky A, Waldner M, Obenauf AC, et al. Spatiotemporal dynamics of intratumoral immune cells reveal the immune landscape in human cancer. Immunity. 2013;39(4):782-95.

15. Spranger S, Luke JJ, Bao R, Zha Y, Hernandez KM, Li Y, et al. Density of immunogenic antigens does not explain the presence or absence of the T-cell-inflamed tumor microenvironment in melanoma. Proc Natl Acad Sci U S A. 2016;113(48):E7759-E68.

16. Alistar A, Chou JW, Nagalla S, Black MA, D'Agostino R, Jr., Miller LD. Dual roles for immune metagenes in breast cancer prognosis and therapy prediction. Genome medicine. 2014;6(10):80.

17. Ayers M, Lunceford J, Nebozhyn M, Murphy E, Loboda A, Kaufman DR, et al. IFN-gamma-related mRNA profile predicts clinical response to PD-1 blockade. J Clin Invest. 2017;127(8):2930-40.

18. Rooney MS, Shukla SA, Wu CJ, Getz G, Hacohen N. Molecular and genetic properties of tumors associated with local immune cytolytic activity. Cell. 2015;160(1-2):48-61.

19. Moffitt RA, Marayati R, Flate EL, Volmar KE, Loeza SG, Hoadley KA, et al. Virtual microdissection identifies distinct tumor- and stroma-specific subtypes of pancreatic ductal adenocarcinoma. Nat Genet. 2015;47(10):1168-78.

20. Chakravarthy A, Khan L, Bensler NP, Bose P, De Carvalho DD. TGF-beta-associated extracellular matrix genes link cancer-associated fibroblasts to immune evasion and immunotherapy failure. Nature communications. 2018;9(1):4692.

21. Yaddanapudi K, Rendon BE, Lamont G, Kim EJ, Al Rayyan N, Richie J, et al. MIF Is Necessary for Late-Stage Melanoma Patient MDSC Immune Suppression and Differentiation. Cancer Immunol Res. 2016;4(2):101-12.

22. Calon A, Espinet E, Palomo-Ponce S, Tauriello DV, Iglesias M, Cespedes MV, et al. Dependency of colorectal cancer on a TGF-beta-driven program in stromal cells for metastasis initiation. Cancer cell. 2012;22(5):571-84.

23. Hanzelmann S, Castelo R, Guinney J. GSVA: gene set variation analysis for microarray and RNA-seq data. BMC bioinformatics. 2013;14:7.
