# Supplementary material for: Identification of cross-talk between m6A and 5mC regulators associated with onco-immunogenic features and prognosis across 33 cancer types
Source: J Hematol Oncol. 2020 Mar 18;13:22. doi: 10.1186/s13045-020-00854-w (PMC7081591; doi:10.1186/s13045-020-00854-w)
Supplement: Supplementary file 6 — Additional file 6: Table S1. Details of the 33 cancer types from the TCGA. [file 13045_2020_854_MOESM6_ESM.docx]

**Table S1. Details of the 33 cancer types from the TCGA**

| **Cancer type** | **Abbreviation** | **Number of samples** |
| --- | --- | --- |
| Adrenocortical carcinoma | ACC | 91 |
| Bladder urothelial carcinoma | BLCA | 412 |
| Breast cancer | BRCA | 1,087 |
| Cervical squamous cell carcinoma and endocervical adenocarcinoma | CESC | 306 |
| Cholangiocarcinoma | CHOL | 36 |
| Colon adenocarcinoma | COAD | 445 |
| Lymphoid neoplasm diffuse large B-cell lymphoma | DLBC | 50 |
| Esophageal carcinoma | ESCA | 183 |
| Glioblastoma multiforme | GBM | 599 |
| Head and neck squamous carcinoma | HNSC | 523 |
| Kidney chromophobe | KICH | 65 |
| Kidney renal clear cell carcinoma | KIRC | 518 |
| Acute myeloid leukemia | LAML | 286 |
| Kidney renal papillary cell carcinoma | KIRP | 200 |
| Brain low-grade glioma | LGG | 514 |
| Liver hepatocellular carcinoma | LIHC | 374 |
| Lung adenocarcinoma | LUAD | 575 |
| Lung squamous cell carcinoma | LUSC | 490 |
| Mesothelioma | MESO | 87 |
| Ovarian serous cystadenocarcinoma | OV | 600 |
| Pancreatic adenocarcinoma | PAAD | 184 |
| Pheochromocytoma and paraganglioma | PCPG | 178 |
| Prostate adenocarcinoma | PRAD | 495 |
| Rectal adenocarcinoma | READ | 159 |
| Sarcoma | SARC | 257 |
| Skin cutaneous melanoma | SKCM | 470 |
| Stomach adenocarcinoma | STAD | 440 |
| Testicular germ cell tumor | TGCT | 149 |
| Thyroid carcinoma | THCA | 503 |
| Thymoma | THYM | 124 |
| Uterine corpus endometrial carcinoma | UCEC | 543 |
| Uterine carcinosarcoma | UCS | 57 |
| Uveal melanoma | UVM | 80 |
