# Supplementary material for: Identification of cross-talk between m6A and 5mC regulators associated with onco-immunogenic features and prognosis across 33 cancer types
Source: J Hematol Oncol. 2020 Mar 18;13:22. doi: 10.1186/s13045-020-00854-w (PMC7081591; doi:10.1186/s13045-020-00854-w)
Supplement: Supplementary file 7 — Additional file 7: Table S2. Immuno-stromal signatures used in the current study. [file 13045_2020_854_MOESM7_ESM.docx]

**Table S2. Immuno-stromal signatures used in the current study**

| **Signature name** | **Reference** | **Suppl. Reference** |
| --- | --- | --- |
| Immune enrichment score | Yoshihara et al. Nat Commun. 2013 | (1) |
| Immune cell subsets | The Cancer Genome Atlas Network. Cell. 2015 | (2) |
| Immune signaling molecules | The Cancer Genome Atlas Network. Cell. 2015 | (2) |
| Immunophenoscore | Charoentong et al. Cell Rep. 2017 | (3) |
| T cells | Bindea et al. Immunity. 2013 | (4) |
| 13-gene T-cell signature | Spranger et al. Proc Natl Acad Sci U S A. 2016 | (5) |
| CD8 T cells | Bindea et al. Immunity. 2013 | (4) |
| T.NK. metagene | Alistar et al. Genome Med. 2014 | (6) |
| B.P. metagene | Alistar et al. Genome Med. 2014 | (6) |
| Macrophages | Bindea et al. Immunity. 2013 | (4) |
| Cytotoxic cells | Bindea et al. Immunity. 2013 | (4) |
| 6-gene IFN-γ signature | Ayers et al. J Clin Invest. 2017 | (7) |
| Type I IFN Response | Rooney et al. Cell. 2015 | (8) |
| Type II IFN Response | Rooney et al. Cell. 2015 | (8) |
| Cytolytic activity | Rooney et al. Cell. 2015 | (8) |
| Activated stroma | Moffitt et al. Nat Genet. 2015 | (9) |
| ECM | Chakravarthy et al. Nat Commun. 2018 | (10) |
| MDSC | Yaddanapudi et al. Cancer Immunol Res. 2016 | (11) |
| CAF | Calon et al. Cancer Cell. 2012 | (12) |

Abbreviations: CAF, cancer-associated fibroblast; ECM, extracellular matrix; IFN, interferon; MDSC, myeloid-derived suppressor cell.

**Supplementary references**

1. Yoshihara K, Shahmoradgoli M, Martinez E, Vegesna R, Kim H, Torres-Garcia W, et al. Inferring tumour purity and stromal and immune cell admixture from expression data. Nature communications. 2013;4:2612.

2. Cancer Genome Atlas N. Genomic Classification of Cutaneous Melanoma. Cell. 2015;161(7):1681-96.

3. Charoentong P, Finotello F, Angelova M, Mayer C, Efremova M, Rieder D, et al. Pan-cancer Immunogenomic Analyses Reveal Genotype-Immunophenotype Relationships and Predictors of Response to Checkpoint Blockade. Cell reports. 2017;18(1):248-62.

4. Bindea G, Mlecnik B, Tosolini M, Kirilovsky A, Waldner M, Obenauf AC, et al. Spatiotemporal dynamics of intratumoral immune cells reveal the immune landscape in human cancer. Immunity. 2013;39(4):782-95.

5. Spranger S, Luke JJ, Bao R, Zha Y, Hernandez KM, Li Y, et al. Density of immunogenic antigens does not explain the presence or absence of the T-cell-inflamed tumor microenvironment in melanoma. Proc Natl Acad Sci U S A. 2016;113(48):E7759-E68.

6. Alistar A, Chou JW, Nagalla S, Black MA, D'Agostino R, Jr., Miller LD. Dual roles for immune metagenes in breast cancer prognosis and therapy prediction. Genome medicine. 2014;6(10):80.

7. Ayers M, Lunceford J, Nebozhyn M, Murphy E, Loboda A, Kaufman DR, et al. IFN-gamma-related mRNA profile predicts clinical response to PD-1 blockade. J Clin Invest. 2017;127(8):2930-40.

8. Rooney MS, Shukla SA, Wu CJ, Getz G, Hacohen N. Molecular and genetic properties of tumors associated with local immune cytolytic activity. Cell. 2015;160(1-2):48-61.

9. Moffitt RA, Marayati R, Flate EL, Volmar KE, Loeza SG, Hoadley KA, et al. Virtual microdissection identifies distinct tumor- and stroma-specific subtypes of pancreatic ductal adenocarcinoma. Nat Genet. 2015;47(10):1168-78.

10. Chakravarthy A, Khan L, Bensler NP, Bose P, De Carvalho DD. TGF-beta-associated extracellular matrix genes link cancer-associated fibroblasts to immune evasion and immunotherapy failure. Nature communications. 2018;9(1):4692.

11. Yaddanapudi K, Rendon BE, Lamont G, Kim EJ, Al Rayyan N, Richie J, et al. MIF Is Necessary for Late-Stage Melanoma Patient MDSC Immune Suppression and Differentiation. Cancer Immunol Res. 2016;4(2):101-12.

12. Calon A, Espinet E, Palomo-Ponce S, Tauriello DV, Iglesias M, Cespedes MV, et al. Dependency of colorectal cancer on a TGF-beta-driven program in stromal cells for metastasis initiation. Cancer cell. 2012;22(5):571-84.
